# Relationship Between Effort-Reward Imbalance, Over-Commitment and Occupational Burnout in the General Population: A Prospective Cohort Study

**DOI:** 10.3389/ijph.2023.1606160

**Published:** 2023-10-06

**Authors:** Yara Shoman, Setareh Ranjbar, Marie-Pierre Strippoli, Roland von Känel, Martin Preisig, Irina Guseva Canu

**Affiliations:** ^1^ Department of Occupational and Environmental Health, Unisante, Université de Lausanne, Lausanne, Switzerland; ^2^ Department of Psychiatry, Psychiatric Epidemiology and Psychopathology Research Center, Lausanne University Hospital, University of Lausanne, Lausanne, Switzerland; ^3^ Department of Consultation-Liaison Psychiatry and Psychosomatic Medicine, University Hospital Zürich, University of Zurich, Zurich, Switzerland

**Keywords:** prospective cohort, occupational health, burnout, general population, exposure to work-related stress

## Abstract

**Objectives:** To prospectively investigate the association between Effort-Reward Imbalance (ERI) and over-commitment and the scores of the burnout dimensions over a 4 years follow-up period considering potential confounders.

**Methods:** Data stemmed from CoLaus|PsyCoLaus, a population-based cohort study including 575 participants (mean age 55 years, 50% men). Participants completed the Maslach Burnout Inventory-General Survey, ERI and over-commitment questionnaires at baseline (T1) and after a 4 years follow-up (T2), and provided demographic, behavioral, psychiatric, personality and social support information through self-reported questionnaires and semi-structured interviews. Serially adjusted linear regression models were used.

**Results:** ERI and over-commitment were not associated longitudinally with any of the burnout dimensions when controlling for confounders. One standard deviation increases in the scores of exhaustion, cynicism and professional efficacy were associated with one standard deviation increase in the scores of the same burnout dimensions longitudinally, and these associations were independent of the effects of ERI and over-commitment.

**Conclusion:** Future studies should re-examine the effect of ERI and over-commitment on workers’ burnout, considering the effects of confounders.

## Introduction

The changes in working conditions brought on by technology advancements [1] have led to an increase in work stress, which has become a growing public health concern [2]. Work stress is defined by World Health Organization (WHO) as the reaction of workers to job demands that exceed their knowledge and abilities [3]. The measurement of work stress can be divided into three approaches: 1-assessment of stressors reported by workers, 2-assessment of responses to stress (job strain such as burnout) and 3- assessment of the experience of work stress [4]. The Effort-Reward Imbalance (ERI) reflects the exposure to work stressors such as high work demands justifying employee’s efforts to manage these efforts and reward or recognition of these efforts. The ERI and over-commitment model allows assessing the impact of these exposures on health by examining the imbalance between job demands (extrinsic and intrinsic) and rewards [5]. The ERI model has been shown to be useful for explaining and preventing work-related stress in a systematic review [6] and has been linked to the development of stress-related disorders through pathways such as altered heart rate variability, blood lipids, and metabolic syndrome [7]. Chronic work stress can additionally lead to occupational burnout [8].

Theoretical approaches to burnout can be classified into four categories: individual, interpersonal, organizational, and societal [9]. The interpersonal model suggests that burnout occurs in three phases: exhaustion from job demands, detachment (cynicism), and reduced professional efficacy [10]. The Maslach Burnout Inventory (MBI), a widely used tool for burnout assessment [11, 12], is based on this model. The WHO has recognized burnout as a factor influencing health status in its 11th revision of the international classification of diseases [13] that is linked to both psychological and physical consequences [14]. Although burnout is often described a response to chronic work stressors, it is distinct from work stress [15, 16]. Following the previously mentioned three approaches for measurement of stress at work, ERI could be the stressors reported by workers whereas burnout is the response to these stressors (i.e., strain).

The evidence on the relevance of the ERI model for research on burnout etiology is currently limited, with three reviews [16–18], and 15 original studies, of which 12 were cross-sectional [19–30] and three were prospective cohorts [31–33]. The limitations of cross-sectional studies in determining the temporal relationships between variables and causality between exposure and effect [34], as well as the scarcity of cohort studies are two major gaps in the literature. The cross-sectional design may result in oversimplification and misinterpretation of the relationship between the exposure to work-related stress and burnout, particularly if the impact of confounding factors is not taken into consideration [35]. The selection of covariates was guided by available literature that reports their associations with burnout [17, 18]. We identified two systematic reviews that concluded a negative correlation between age and burnout symptoms [19, 20]. Another meta-analysis revealed a slight inclination for women to experience emotional exhaustion, while men tend to exhibit depersonalization more frequently [21]. The Aronsson et al., meta-analysis recommended including smoking as a covariate when analyzing burnout studies [22]. Notably, a meta-analysis demonstrated a significant association between burnout and depression (*r* = 0.52) as well as burnout and anxiety (*r* = 0.46) [23]. Further insights were gained from a study of the Finnish general working population, where burnout was notably correlated with alcohol dependence [24]. Personality treats such as neuroticism and extraversion were linked to increased exhaustion (rho = 0.33 and 0.13, respectively) in a meta-analysis based on three and one study(s), respectively [18]. Additionally, a systematic review underscored the significance of support from both supervisors and coworkers in preventing burnout among nurses [25]. Higher effort-reward imbalance was reported to be correlated with higher age [26], in men compared to women [27], in smokers compared to non-smokers [28], with less physical activity [29], and more alcohol use [30]. ERI was also reported to predict affective disorders [31] in general [31] and depression specifically [32]. Higher ERI was correlated with lower social support [33] and extraversion and increased neuroticism [34] were also reported in the literature.

Therefore, the impact of these variables on the relationship between dimensions of the ERI model and occupational burnout requires careful consideration.

A third gap in the literature comes with the inconsistencies in previous findings of the three prospective cohort studies mentioned above. Hadžibajramović et al. established a significant relationship between effort, reward, their joint exposure and burnout, after controlling for potential confounding factors such as social support, physical activity, age, and time [35]. Leineweber et al. observed significant direct paths between ERI and exhaustion, as measured three times with a 2 years interval and controlled for age, sex, income, and job change [36]. Conversely, Nuebling et al. found that only reward predicted burnout after adjusting for age, sex, socioeconomic status, and baseline burnout [37].

Therefore, the aims of this study were to 1) investigate the prospective association between the exposure to work-related stress at baseline, modeled as effort, reward and their combined effect, over-commitment, and the three burnout dimensions: exhaustion, cynicism, and professional efficacy across a 4 years follow-up; and 2) test the potentially confounding effects of variables, rigorously identified in the published literature. The Swiss prospective population-based cohort Colaus|PsyCoLaus [38, 39] provides a robust methodological approach to address the limitations of previous studies in investigating the association between the dimensions of the ERI, over-commitment, and occupational burnout, while controlling for potential confounding factors.

We propose six hypotheses:

1) Higher effort-reward imbalance, effort, and over-commitment will be associated with an increase in exhaustion and cynicism longitudinally.
2) Higher reward will be associated with a decrease in exhaustion and cynicism longitudinally.
3) Higher effort-reward imbalance, effort, and over-commitment will be associated with lower professional efficacy longitudinally.
4) Higher reward will be associated with an increase in professional efficacy longitudinally.
5) High scores in burnout dimensions will be associated with increased scores of the same burnout dimensions longitudinally.
6) The associations between effort, reward and their combined effect, over-commitment, and the three burnout dimensions exhaustion, cynicism, and professional efficacy, will decrease after adjustment for potentially confounding effects of other variables.

## Methods

### Study Sample and Follow-Up

**FIGURE 1:**
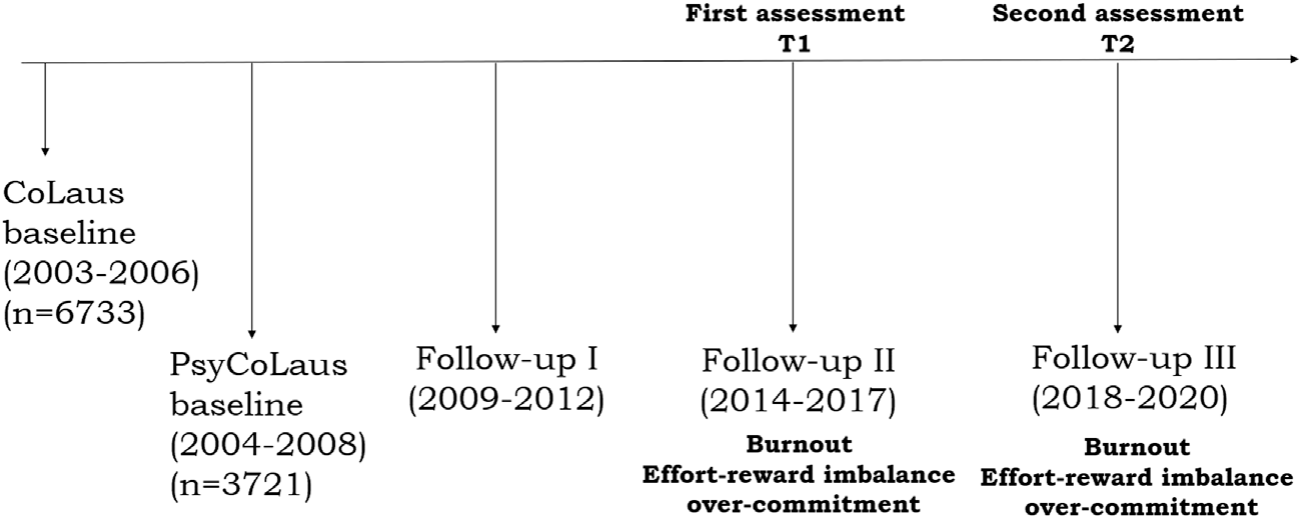
CoLaus|PsyCoLaus follow-ups and variables assessed at each follow-up (CoLaus|PsyCoLaus, Lausanne, Switzerland 2003–2020).

**FIGURE 2:**
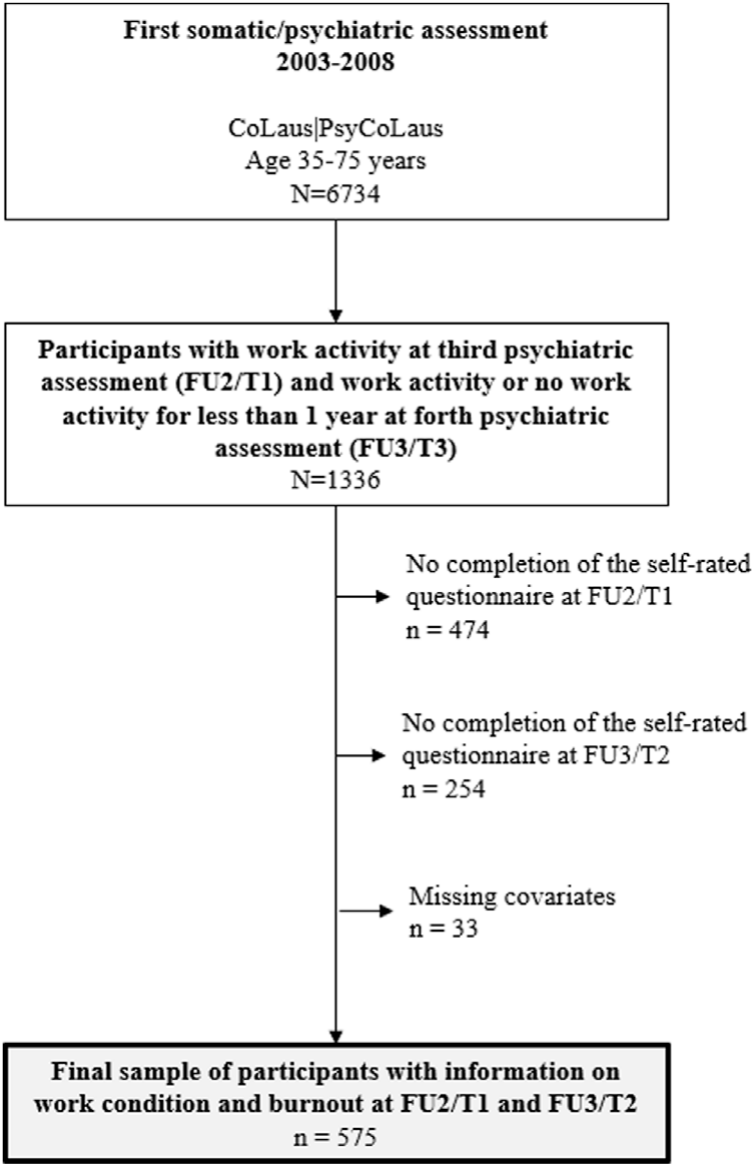
Flow chart of the study for the associations between Effort-Reward Imbalance and occupational burnout in a 4 years follow-up (CoLaus|PsyCoLaus, Lausanne, Switzerland 2003–2020). FU2, follow-up 2; FU3, follow-up 3. T1, first assessment, T2, second assessment.

The present data stem from the prospective cohort study CoLaus|PsyCoLaus, designed to assess cardiovascular risk factors and mental disorders in the community as well as their associations. The methodological features of the recruitment and baseline assessments of CoLaus|PsyCoLaus have been described in detail elsewhere [38, 39]. Briefly, CoLaus|PsyCoLaus includes a random sample of 6,734 participants (age range: 35–75 years) selected from the general population according to the civil register of the city of Lausanne (Switzerland). After a first physical and psychiatric investigation, which took place between 2003 and 2008, the cohort was followed-up for approximately 5 (first follow-up, FU1), 9 (second follow-up, FU2), and 13 years (third follow-up, FU3). Burnout, ERI and over-commitment were only measured at FU2 (first assessment, called T1) and FU3 (second assessment, called T2), the analysis was thus restricted to these two follow-ups (Figure 1). From T1 onward, current workers and participants without occupational activity for a maximum 1 year were invited to fill in self-reported questionnaires on ERI, over-commitment and burnout during the psychiatric evaluation. A total of 1,336 participants reported occupational activity at T1 and occupational activity or retirement for less than 1 year at T2. Among them, 608 participants completed burnout and ERI questionnaires at both T1 and T2. Participants received the self-administered questionnaire during the psychiatric assessment. Two reminders were sent if they did not return the questionnaire. A total of 33 participants had incomplete data on potential confounders and were excluded, resulting in a final sample of 575 participants (Figure 2). A description of the sample is provided in Table 1. Only 6.4% changed their occupational activities between the first (T1) and the second (T2) assessment. The mean (SD) age of the participants was 54.7 (5.3) years and 50% were men. Almost half of the participants were former smokers; the majority reported to be physically active for at least once a week. About half of the participants had Major Depressive Disorders (MDD) or suffered from depression in the past and one-fourth with an anxiety disorder or suffered from anxiety in the past.

**TABLE 1:** Descriptive statistics of the study variables among participants (CoLaus|PsyCoLaus, Lausanne, Switzerland 2003–2020).

	All participants
Number of participants	575
Demographic characteristics	
Age at T1 (years), mean (SD)	54.72 (5.33)
Male sex, %	49.7
Length of follow-up (years), mean (SD)	3.63 (0.52)
Behavioral characteristics at T1	
Smoking status, %	
Active smokers	9.7
Former smokers	48.7
Non smokers	41.6
Physical activity^a^, %	80.2
Lifetime psychiatric disorder until T1	
Major Depressive Disorder, %	
Current	7.3
Recovered	44.3
Never depressed	48.3
Illicit drug use disorder (abuse/dependence), %	
Current	0.3
Recovered	9.2
Never	90.4
Alcohol use disorder (abuse/dependence), %	
Current	2.8
Recovered	11.7
Never	85.6
Anxiety disorder^b^, %	
Current	3.1
Recovered	20.3
Never	76.5
Personality traits at T1, mean (SD)	
Neuroticism	7.81 (5.31)
Extraversion	12.05 (4.77)
Social support at T1, mean (SD)	
From family	5.52 (1.52)
From friends	5.54 (1.43)
From significant others	6.06 (1.24)
Effort-Reward imbalance and over- commitment at T1, mean (SD)	
Effort	12.15 (3.68)
Reward	
Esteem	11.61 (1.90)
Security	6.87 (1.87)
Promotion	8.38 (1.72)
Effort-reward ratio	0.26 (0.12)
Over-commitment	13.69 (3.53)
Burnout at T1	
Exhaustion, mean (SD)	1.50 (1.17)
Cynicism, mean (SD)	1.69 (1.41)
Professional efficacy, mean (SD)	4.64 (0.88)
Burnout at T2	
Exhaustion, mean (SD)	1.57 (1.22)
Cynicism, mean (SD)	1.81 (1.40)
Professional efficacy, mean (SD)	4.66 (0.85)

T1, first assessment; T2, second assessment; SD, standard deviation.

^a^ At least once a week.

^b^ Generalized anxiety disorder, panic disorder, agoraphobia, social phobia.

### Measurement of Study Variables

#### Effort-Reward Imbalance, Over-Commitment, and Burnout

Siegrist proposed a model to assess the exposure to work-related stress through an extrinsic component which is ERI and an intrinsic component which is over-commitment [40]. ERI and over-commitment were measured using the French validated version [41] of Siegrist’s Job Stress Questionnaire [40]. This questionnaire consists of 23 items split in three subscales: effort (*6 items*), reward [i.e., esteem (*4 items*), promotion (*5 items*), and security (*2 items*)], and overcommitment (*6 items*). Examples of items are “Over the past few years, my job has become more and more demanding” from the effort subscale, “Considering all my efforts and achievements, my salary/income is adequate” from the reward subscale and “At work, I am often pressed for time” from the over-commitment subscale. Each item can be scored on a 5-point Likert scale (i.e., from 1 “Strongly disagree” to 4 “Strongly agree”) in the effort and reward subscales, and on a 5-point Likert scale in the over-commitment subscale. The effort/reward ratio is calculated using the formula: effort/reward*correction factor, where the correction factor (0.55) adjusts for the unequal number of items of the effort and reward scores [42]. The Cronbach’s alphas of all subscales were reported to be higher than 0.70: 0.75 for the effort subscale, 0.86 (for men) and 0.88 (for women) for the reward subscale and 0.82 for the over-commitment subscale.

Burnout was measured using the French validated version [43] of the Maslach Burnout Inventory General Survey (MBI-GS) [44]. The 16 items of the MBI-GS are scored on a 7-point Likert-type scale from 0 “never” to 6 “every day”. This questionnaire consists of two negatively worded subscales; *exhaustion* (*5 items*), and *cynicism* (*5 items*) and one positively worded subscale; *professional efficacy* (*6 items*). Examples of items are “I feel burned out from my work” from the exhaustion subscale, “I would just like to do my job without being disturbed” from the cynicism subscale and “I think I am good at my job” from the professional efficacy subscale. The Cronbach’s alphas of all subscales were reported to be higher than 0.70: 0.90 for the exhaustion subscale, 0.86 for the cynicism subscale and 0.87 for the professional efficacy subscale.

#### Potential Confounders

Diagnostic information on mental disorders was collected using the French version [45] of the semi-structured Diagnostic Interview for Genetic Studies (DIGS) [46]. The French version of this instrument has adequate inter-rater and test-retest reliability for major mood [47] and substance use disorders [48]. The DIGS was completed with anxiety disorder sections of the French version [49] of the Schedule for Affective Disorders and Schizophrenia-Lifetime and Anxiety disorder version (SADS-LA) [50]. Diagnoses were assigned according to the Diagnostic and Statistical Manual of Mental Disorders (DSM-IV) [51]. The DIGS also collects information on smoking status and physical activity (at least once a week). Interviewers were master-level psychologists, who were trained over a 1–2 months period. Each interview and diagnostic assignment was reviewed by an experienced senior psychologist.

The personality dimensions of Neuroticism and Extraversion were assessed using the French validated version [52] of the Eysenck Personality Questionnaire (EPQ) [53]. Social support from family, friends, and significant others was measured using the French version [54] of the Multidimensional Scale of Perceived Social Support (MSPSS) [55].

The potential confounders were age, sex, physical activity, smoking status, psychiatric disorders [major depressive disorder, anxiety disorders (generalized anxiety disorder, panic disorder, agoraphobia, social phobia), illicit drug and alcohol use disorders (abuse or dependence)], personality traits (neuroticism and extraversion), and social support (from friends, family and significant others).

### Statistical Analysis

Data descriptive for demographic feature, health behaviors, psychiatric disorders, personality traits and social support were used to characterize the sample. In order to assess the associations between the ERI and over-commitment dimensions at T1 and the score of the three burnout dimensions (exhaustion, cynicism, professional efficacy) at T2, two separate sets of serially adjusted linear regression models were run: one set of models included the effort-reward ratio and the over-commitment baseline dimensions as independent variables, the other set of models included the ERI baseline dimensions [effort, the three reward sub-dimensions (esteem, promotion and security)] as well as the over-commitment baseline dimension as independent variables. The scores of the three burnout dimensions at T2 were the dependent variables. The first model (Model 1) was adjusted for the respective burnout dimension at T1. Model 2 was additionally adjusted for age, sex, the follow-up duration and the two other burnout dimension scores at T1. The fully adjusted model (Model 3) was the Model 2 further adjusted for potential confounders at T1 [physical activity, smoking status, psychiatric disorders (major depressive disorder, anxiety disorders (generalized anxiety disorder, panic disorder, agoraphobia, social phobia), illicit drug and alcohol use disorders (abuse or dependence)), personality traits, and social support]. We computed standardized beta coefficients by transforming all continuous variables to z-scores. Prior to conducting our analysis, we assessed for multicollinearity among the variables and found that none of the correlations exceeded a threshold of *r* = 0.70 [56], and the highest observed correlation was 0.62 (Supplementary Table S1). We performed paired t-tests to compare the scores of burnout dimensions between T1 and T2. The missing data in this study was 5% for all variables including confounders. Multiple imputation provides negligible benefit in this case [57, 58], we thus decided to perform a list-wise exclusion and complete case analysis. All statistical analyses were performed using Stata statistical software version 16 [59] and a *p*-value <0.05 was considered statistically significant.

## Results

### The Associations Between Effort-Reward Imbalance, Over-Commitment, and the Dimensions of Burnout

Using the first set of models with the effort-reward ratio and the over-commitment dimension at the first assessment as independent variables, no significant associations were found between these scores and the three burnout dimensions scores at T2 (Models 1, 2, 3, Table 2). Using the second set of models with the ERI (Effort, esteem, promotion, security) and the over-commitment dimensions at the first assessment as independent variables, one standard deviation increase in promotion scores at T1 was significantly associated with one standard deviation decrease in the scores of exhaustion at T2 (Model 1, Table 2). Nevertheless, this association became non-significant in Models 2 and 3 after adjustment for potential confounders. One standard deviation increase in the scores of security and promotion at T1 were associated with one standard deviation decrease in the scores of cynicism at T2 in Models 1 and 2. However, these associations became non-significant in the fully adjusted model (Model 3, Table 2) taking into account health behaviors, psychiatric disorder, personality traits and social support. Effort, esteem and over-commitment dimensions were not associated with any scores of burnout dimensions in this second set of analyses. The Akaike Information Criterion (AIC) for the models 1, 2, and 3 indicated that Model 3 performance is the most preferable for exhaustion and cynicism whereas Model 2 performance is the most preferable for professional efficacy (Table 2).

**TABLE 2:** Linear regression models of the associations between the Effort-Reward Imbalance (ERI) dimensions, effort-reward ratio, and over-commitment at the first assessment and the scores of burnout dimensions at the second assessment with 4 year follow-up period (*n* = 575) (CoLaus|PsyCoLaus, Lausanne, Switzerland 2003–2020).

ERI and over-commitment at the first assessment	Exhaustion			Cynicism			Professional efficacy		
	Model 1^a^	Model 2^b^	Model 3^c^	Model 1^a^	Model 2^b^	Model 3^c^	Model 1^a^	Model 2^b^	Model 3^c^
	β (95% CI)	Β (95% CI)	β (95% CI)	β (95% CI)	β (95% CI)	β (95% CI)	β (95% CI)	β (95% CI)	β (95% CI)
Effort-reward ratio	0.02 (−0.06, 0.11)	0.01 (−0.08, 0.10)	−0.0004 (−0.09, 0.09)	0.09 (−0.002, 0.18)	0.06 (−0.03, 0.16)	0.07 (−0.02, 0.16)	−0.06 (−0.15, 0.02)	0.02 (−0.07, 0.12)	0.03 (−0.06, 0.13)
Over-commitment	0.05 (−0.03, 0.13)	0.05 (−0.04, 0.13)	0.02 (−0.06, 0.10)	0.03 (−0.05, 0.11)	0.00001 (−0.09, 0.09)	−0.02 (−0.11, 0.06)	0.01 (−0.07, 0.09)	0.05 (−0.03, 0.14)	0.06 (−0.03, 0.15)
Adjusted *R* ^2^	*0.31*	*0.31*	*0.35*	*0.24*	*0.26*	*0.28*	*0.24*	*0.26*	*0.26*
AIC	*1422.381*	*1425.948*	*1405.825*	*1483.816*	*1477.42*	*1476.862*	*1479.473*	*1466.317*	*1480.779*
Effort	−0.004 (−0.10, 0.09)	−0.02 (−0.11, 0.07)	0.004 (−0.09, 0.10)	0.07 (−0.03, 0.16)	0.05 (−0.05, 0.15)	0.07 (−0.02, 0.17)	0.03 (−0.06, 0.12)	0.09 (−0.01, 0.18)	0.08 (−0.02, 0.18)
Reward									
Esteem	0.02 (−0.06, 0.09)	0.02 (−0.06, 0.09)	0.03 (−0.05, 0.10)	0.07 (−0.01, 0.15)	0.07 (−0.01, 0.15)	0.08 (−0.00, 0.16)	0.03 (−0.05, 0.11)	0.003 (−0.08, 0.08)	0.01 (−0.07, 0.09)
Security	−0.003 (−0.08, 0.07)	−0.02 (−0.09, 0.07)	0.01 (−0.07, 0.09)	**−0.10 (−0.19, −0.02)**	**−0.08 (−0.17, −0.002)**	−0.07 (−0.15, 0.01)	0.07 (−0.01, 0.15)	0.04 (−0.04, 0.12)	0.02 (−0.07, 0.10)
Promotion	**−0.08 (−0.16, −0.01)**	−0.06 (−0.14, 0.01)	−0.04 (−0.12, 0.03)	**−0.08 (−0.15, 0.002)**	**−0.08 (−0.16, −0.003)**	−0.06 (−0.14, 0.02)	0.03 (−0.04, 0.11)	0.002 (−0.8, 0.08)	−0.004 (−0.08, 0.08)
Over-commitment	0.06 (−0.03, 0.15)	0.06 (−0.02, 0.15)	0.02 (−0.06, 0.11)	0.01 (−0.08, 0.10)	−0.01 (−0.10, 0.08)	−0.04 (−0.13, 0.05)	−0.01 (−0.10, 0.08)	0.03 (−0.06, 0.12)	0.03 (−0.06, 0.13)
Adjusted *R* ^2^	*0.31*	*0.32*	*0.35*	*0.25*	*0.27*	*0.28*	*0.24*	*0.26*	*0.26*
AIC	*1423.48*	*1421.255*	*1410.249*	*1477.91*	*1472.975*	*1473.806*	*1481.301*	*1468.855*	*1484.327*

95CI, 95% confidence interval; ERI, Effort-Reward ratio; AIC, akaike information criterion.

^a^ Model 1 is adjusted for the outcome at the first assessment.

^b^ Model 2 is adjusted for the outcome at the first assessment, age at the first assessment, sex, length between the first and second assessments, and other burnout dimensions at the first assessment (exhaustion, cynicism, professional efficacy).

^c^ Model 3 is adjusted for covariates in Model 2 plus, smoking status and physical activity at the first assessment, personality traits (neuroticism, extraversion), social support (friends, family, significant others) and lifetime psychiatric disorders (major depressive disorder, substance use disorders, anxiety disorders) at the first assessment.

Statistically significant results are in bold.

### The Associations Between Potential Confounders and Burnout

The results of paired t-test indicated that the score of exhaustion (*p* = 0.004) and cynicism (*p* = 0.02) were lower at T1 compared to T2. The scores of professional efficacy were not statically different (*p* = 0.45). In the fully adjusted models (Models 3) including either the effort-reward ratio and the over-commitment dimensions or the ERI (effort, esteem, promotion, security) and the over-commitment dimensions at the first assessment as independent variables, one standard deviation increase in age was associated with a one standard deviation decrease in exhaustion scores at T2 while one standard deviation increase in neuroticism score was associated with a one standard deviation increase in exhaustion scores at T2 (Table 3 and Supplementary Table S2). In addition, when using the effort-reward ratio and the over-commitment dimensions at the first assessment as independent variables, one standard deviation increase in extraversion scores was associated with a one standard deviation decrease in exhaustion scores at the second assessment (Table 3). Similarly, using either the effort-reward ratio and the over-commitment dimensions or the ERI and the over-commitment dimensions at the first assessment as independent variables, being physically active for at least once per week and a one standard deviation increase in extraversion scores at T1 were associated with a one standard deviation decrease in cynicism scores at T2 (Table 3 and Supplementary Table S2). One standard deviation increase in scores of exhaustion, cynicism and professional efficacy at T1 were associated with a one standard deviation increase in the scores of the same burnout dimensions at T2, and these associations were independent of the effects of ERI and over-commitment. In addition, a one standard deviation increase in cynicism score at T1 was associated with a one standard deviation decrease in professional efficacy scores at T2 (Table 3 and Supplementary Table S2). No significant associations between sex, smoking status, lifetime psychiatric disorders (major depressive disorder, substance use disorders, and anxiety disorders), social support (friends, family, significant others) at T1 and the scores of exhaustion, cynicism, and professional efficacy at T2 were found.

**TABLE 3:** Fully adjusted linear regression models of the associations between Effort-Reward Imbalance (ERI) dimensions, effort-reward ratio, and over-commitment measured at the first assessment (2014–2018) and the scores of burnout dimensions measured at the second assessment (2018–2021) adjusting for potential covariates (*n* = 575) (CoLaus|PsyCoLaus, Lausanne, Switzerland 2003–2020).

	Exhaustion	Cynicism	Professional efficacy
	β (95% CI)	β (95% CI)	β (95% CI)
Effort-reward ratio	−0.00 (−0.09, 0.09)	0.07 (−0.02, 0.16)	0.03 (−0.06, 0.13)
Over-commitment	0.02 (−0.06, 0.10)	−0.02 (−0.11, 0.06)	0.06 (−0.03, 0.15)
Age	**−0.12 (−0.19, −0.05)**	0.01 (−0.06, 0.08)	−0.01 (−0.08, 0.07)
Sex	−0.06 (−0.21, 0.09)	0.13 (−0.03, 0.29)	0.11 (−0.05, 0.27)
Length of follow-up	−0.06 (−0.13, 0.01)	−0.02 (−0.09, 0.05)	0.03 (−0.04, 0.11)
Smoking status			
Non smokers	References		
Active smokers	0.14 (−0.01, 0.29)	0.09 (−0.07, 0.25)	−0.05 (−0.21, 0.11)
Former smokers	0.07 (−0.18, 0.32)	−0.08 (−0.34, 0.18)	−0.08 (−0.35, 0.18)
Physical activity^a^	−0.15 (−0.02, 0.32)	**−0.22 (0.03, 0.40)**	0.05 (−0.23, 0.13)
Burnout at T1			
Exhaustion	**0.43 (0.32, 0.53)**	0.10 (−0.01, 0.21)	−0.02 (−0.13, 0.09)
Cynicism	0.01 (−0.08, 0.10)	**0.32 (0.22, 0.41)**	**−0.16 (**−**0.26,** −**0.06)**
Professional efficacy	0.04 (−0.03, 0.11)	−0.03 (−0.11, 0.05)	**0.42 (0.35, 0.50)**
Major Depressive Disorder			
Never depressed	References		
Current	0.02 (−0.13, 0.29)	−0.01 (−0.17, 0.15)	0.01 (−0.15, 0.17)
Recovered	−0.22 (−0.18, 0.32)	0.06 (−0.25, 0.37)	0.03 (−0.29, 0.34)
Illicit drug use disorder (abuse/dependence)			
Never	References		
Current	0.15 (−0.09, 0.39)	0.07 (−0.18, 0.33)	0.17 (−0.08, 0.43)
Recovered	−0.61 (−1.78, 0.56)	−0.40 (−1.64, 0.85)	0.38 (−0.87, 1.63)
Alcohol use disorder (abuse/dependence)			
Never	References		
Current	0.08 (−0.14, 0.30)	0.15 (−0.08, 0.39)	−0.06 (−0.29, 0.18)
Recovered	0.14 (−0.27, 0.55)	−0.19 (−0.63, 0.25)	0.28 (−0.16, 0.72)
Anxiety disorders^b^			
Never	References		
Current	0.04 (−0.13, 0.22)	−0.09 (−0.28, 0.09)	−0.04 (−0.23, 0.14)
Recovered	−0.27 (−0.67, 0.13)	−0.09 (−0.51, 0.33)	0.19 (−0.24, 0.61)
Personality traits			
Neuroticism	**0.16 (0.08, 0.25)**	0.08 (−0.01, 0.18)	−0.07 (−0.17, 0.01)
Extraversion	**−0.07 (−0.15, 0.00)**	**−0.09 (−0.17, −0.01)**	0.04 (−0.04, 0.12)
Social support			
From family	0.02 (−0.07, 0.10)	0.02 (−0.07, 0.11)	−0.08 (−0.17, 0.01)
From friends	0.01 (−0.08, 0.09)	−0.06 (−0.14, 0.03)	0.04 (−0.05, 0.13)
From significant others	−0.08 (−0.16, 0.01)	−0.05 (−0.14, 0.05)	0.08 (−0.01, 0.17)
Adjusted R^*2*^	*0.35*	*0.28*	*0.26*

Statistically significant results are in bold

^a^ At least once a week.

^b^ Generalized anxiety disorder, panic disorder, agoraphobia, social phobia.

## Discussion

### Main Findings

Based on a large population-based prospective cohort, the most salient findings were: 1) a higher score in the reward sub-scale promotion at the first assessment were associated with a decrease in exhaustion and cynicism scores, and a higher security score at the first assessment was associated with a decrease of cynicism score across a 4 years follow-up period with minimal adjustment; 2) after controlling for potential confounding factors, these associations did not reach statistical significance. Age, personality traits (neuroticism and extraversion) and physical activity were associated with exhaustion or cynicism scores across the follow-up period.

The scores of exhaustions and cynicism were higher at the second assessment compared to the first assessment. Since the second assessment was performed between 2018 and 2020, this may be partially explained by COVID-19 pandemic. Indeed, the pandemic increased the exposure to work-related psychosocial factors which led to the increase in burnout. Due to the lack of a valid cut-off for MBI-GS, we could not investigate whether the workers had an increased rate of exhaustion and cynicism, after the 4 years follow-up but they did not develop burnout yet or they developed burnout and continued working.

Finally, a higher score of a burnout dimension at the first assessment was associated with an increase in the score of the same burnout dimension after a 4 years follow-up with the largest effect sizes compared to the independent variables and other confounders. This finding indicates that the changes in the outcomes between the first and the second assessment are largely explained by the outcome scores at the first assessment. In addition, cynicism at the first assessment was associated longitudinally with a decrease in the score of professional efficacy. This finding is in line with the argument stated by Leiter and Maslach that exhaustion is the first burnout symptom to develop, leading then to cynicism or distancing from work as a maladaptive coping strategy, which, if not managed appropriately, can further lead to reduced professional efficacy [60].

### Effort-Reward Imbalance, Over-Commitment and Burnout

The results from three previous cohort studies [35–37], in which burnout was measured using the Shirom-Melamed Burnout Measure (SMBM) [61] or the Copenhagen Burnout Inventory (CBI) [62], showed associations between ERI and burnout. However, in the study of Nuebling et al., 2022, only reward remained to be significantly associated with burnout after adjustment for the outcome at baseline although the strength of this association was three-fold smaller. We also controlled for the outcomes at the first assessment in our study and the only statistically significant associations were the one between promotion and exhaustion and the ones between promotion, security and cynicism before controlling for all confounders. The inclusion of a large set of confounders in the fully adjusted models (Model 3), may explain why initially identified associations turned statistically non-significant. The cross-sectional studies previously conducted in Switzerland [63–69], found that the exposure to work-related stress measured using ERI predicts burnout. Some of these studies concluded that the association between ERI and burnout is weaker compared to that with work-life balance [66], work-privacy conflict [68], or neuroticism [63]. In the present study, we also found a significant effect of neuroticism on an increase in exhaustion. This study is the first one conducted in Switzerland that examined the relationship between over-commitment and the dimensions of burnout. However, beyond Switzerland, over-commitment was associated with burnout in two longitudinal studies [37, 70] and with the three burnout dimensions measured using the MBI-GS, in two cross-sectional studies [71, 72]. In the four aforementioned studies, over-commitment was associated with an increase in burnout and in its three dimensions.

### The Effects of Other Confounders

Age range may have influenced the results of this study since all participants were older than 47 years. Two systematic reviews concluded that young age is associated with higher rates of burnout symptoms [19, 20, 73]. Among the other confounders, we found a negative association of physical activity with cynicism but not with exhaustion This contradicts the results of prior studies reviewed by [74]. We examined the association between two personality traits (i.e., neuroticism, and extraversion) and burnout. Two cross-sectional studies in Switzerland also found that neuroticism is associated with an increase in burnout while extraversion is associated with a decrease in burnout [63, 64], and the effect size of the association with neuroticism was also larger than the one with extraversion; these findings confirm the results of our study. It is worth mentioning that neuroticism and exhaustion are overlapping constructs, so when both are measured using self-reported questionnaires, negative affectivity may have biased the results [9]. We found no association between social support and burnout, opposite to what was suggested in two out of three cross-sectional studies addressing this question in Switzerland [64, 68]. The differences between the results of this study and those previously cited, may be potentially attributed to the nature of the analysis employed (i.e., longitudinal versus cross-sectional).

It is noteworthy to mention that the prevalence of psychiatric disorders was elevated in this study sample, although this sample is a sample of the general population. The reason for this high prevalence could be clarified by the specific features of the PsyCoLaus sample and the diagnostic instrument used [75]. First, the PsyCoLaus sample was recruited from an urban area (the city of Lausanne) where there is evidence for high prevalence of psychiatric disorder. Second, the features of the diagnostic instrument (DIGS interview) further encouraged the high estimates of psychiatric disorders. Lastly, we cannot rule out that participants with chronic forms of psychiatric disorders were more likely to take part in the study [75].

### Strengths and Limitations

The present study is a pioneering cohort study in Switzerland and one of the few in Europe to investigate the longitudinal relationship between the exposure to work-related stress, as modeled by the ERI, over-commitment, and the dimensions of burnout. To control for potential confounding variables, physician-assessed diagnoses of Major Depressive Disorder, anxiety disorders, alcohol use disorder, and drug use disorder were included in the models. Additionally, baseline burnout dimensions scores were controlled for in the analysis of the longitudinal associations over a 4 years follow-up period. However, the study also has some limitations. Self-reported measurement tools were utilized to assess ERI, over-commitment, burnout, support, and personality traits, which may have influenced their results by negative affectivity [76] or social desirability [77]. The source population consisted of the randomly selected residents of Lausanne, Switzerland and therefore, caution should be exercised when generalizing the results to other regions in Switzerland or other countries.

### Implication of the Findings

ERI and over-commitment were not associated longitudinally with any of the burnout dimensions when other potentially confounding factors (age, sex, physical activity, smoking status, psychiatric disorders, personality traits, and social support) were taken into account. These findings are different compared to cross-sectional studies or longitudinal ones with no adequate control for confounding factors. While this study has numerous advantages, specifically the certainty of assessment of temporal effects (in contrast to cross-sectional studies), causal assumptions should not be drawn considering there are other important criteria of causality that were not met [78]. The study design implemented in this study is observational and not experimental and thus some criteria could not be tested particularly with merely two measurement points. Nonetheless, prospective cohort studies have the strongest potential to identify a causal risk factor among observational studies.

The findings of this study hold important implications, particularly in the context of the Swiss Federal Office of Statistics’ report from the Swiss Health Survey of 2017, which indicated that the Lake Geneva region had a high prevalence of emotional exhaustion and work stress [73]. Thus, our study adds valuable information on the relationship between the exposure to work-related stress and occupational burnout in the working population of Lausanne.

#### Practical Implications

Based on the findings of this study we suggest future studies to re-examine the associations between the exposure to work stress and burnout in the activity sectors with high prevalence of emotional exhaustion and work stress [73]. Future studies should also use shorter follow-up periods (less than 1 year) between measurement points and control for known confounders. Burnout latency may be relatively short (i.e., 6–12 months) followed by a potential recovery or uncontrolled bias. Another suggestion could be to implement future studies using shorter follow-up lengths (up to 1 year) between measurement points while taking the effects of confounders into account. Burnout latency may be relatively short based on the finding of a meta-analysis of burnout predictors [18].

The results of this study underline the need for future studies focused on younger workers and targeted interventions to reduce occupational burnout. Additionally, promoting intergenerational collaboration in the workplace may also prove useful in mitigating burnout since older workers may help younger ones in developing adaptive coping strategies. Older workers were shown to have better skills in stress management at work and report less burnout compared to young workers [79, 80].
