# Corrigendum: Relationship Between Effort-Reward Imbalance, Over-Commitment and Occupational Burnout in the General Population: A Prospective Cohort Study

**DOI:** 10.3389/ijph.2024.1607430

**Published:** 2024-05-16

**Authors:** Yara Shoman, Setareh Ranjbar, Marie-Pierre Strippoli, Roland von Känel, Martin Preisig, Irina Guseva Canu

**Affiliations:** ^1^ Department of Occupational and Environmental Health, Unisante, Université de Lausanne, Lausanne, Switzerland; ^2^ Department of Psychiatry, Psychiatric Epidemiology and Psychopathology Research Center, Lausanne University Hospital, University of Lausanne, Lausanne, Switzerland; ^3^ Department of Consultation-Liaison Psychiatry and Psychosomatic Medicine, University Hospital Zürich, University of Zurich, Zurich, Switzerland

**Keywords:** prospective cohort, occupational health, burnout, general population, exposure to work-related stress

In the original article, there was an error.

A correction has been made to **Methods**, Measurement of Study Variables, Effort-Reward Imbalance, Over-Commitment, and Burnout, Paragraph 1. This sentence previously stated:

“This questionnaire consists of 23 items split in three subscales: effort (6 items), reward [i.e., esteem (5 items), promotion (1 items), and security (5 items)], and over-commitment (6 items).”

The corrected sentence appears below:

“This questionnaire consists of 23 items split in three subscales: effort (6 items), reward [i.e., esteem (4 items), promotion (5 items), and security (2 items)], and over-commitment (6 items).”

The authors apologize for this error and state that this does not change the scientific conclusions of the article in any way. The original article has been updated.

