# Supplementary material for: Relationship Between Effort-Reward Imbalance, Over-Commitment and Occupational Burnout in the General Population: A Prospective Cohort Study
Source: Int J Public Health. 2023 Oct 6;68:1606160. doi: 10.3389/ijph.2023.1606160 (PMC10587427; doi:10.3389/ijph.2023.1606160)
Supplement: Supplementary file 1 [file DataSheet1.pdf]

Supplementary Table S1. Correlation matrix between study variables (n=575)

	1	2	3	4	5	6	7	8	9	10	11	12	13	14	15	16	17	18
**1. Age**	1																	
**2. Effort-Reward ratio**	-0.10	1																
**3. Effort**	-0.12	0.81	1															
**4. Security**	-0.002	-0.61	-0.37	1														
**5. Esteem**	0.01	-0.43	-0.05	0.22	1													
**6.Promotion**	0.10	-0.34	-0.05	0.22	0.37	1												
**7. Over-commitment**	-0.04	0.50	0.59	-0.32	-0.09	-0.04	1											
**8. Length of follow-up**	-0.05	-0.03	0.0003	0.05	0.03	0.08	-0.07	1										
**9. Neuroticism**	-0.07	0.32	0.28	-0.30	-0.13	-0.18	0.40	0.001	1									
**19. Extraversion**	-0.01	-0.15	-0.07	0.17	0.12	0.13	-0.11	0.004	-0.23	1								
**11. Social support from family**	-0.03	-0.20	-0.11	0.15	0.19	0.18	-0.16	0.05	-0.29	0.24	1							
**12. Social support from friends**	-0.06	-0.19	-0.15	0.19	0.13	0.12	-0.16	-0.01	-0.21	0.30	0.38	1						
**13. Social support from significant others**	-0.03	-0.17	-0.13	0.18	0.13	0.11	-0.08	-0.02	-0.21	0.22	0.55	0.51	1					
**14. Exhaustion (T1)**	-0.14	0.54	0.53	-0.35	-0.16	-0.17	0.48	-0.05	0.52	-0.21	-0.30	-0.31	-0.24	1				
**15. Cynicism (T1)**	-0.09	0.50	0.40	-0.40	-0.27	-0.24	0.34	-0.05	0.39	-0.22	-0.26	-0.27	-0.20	0.62	1			
**16. Professional efficacy (T1)**	-0.0003	-0.09	0.01	0.15	0.13	0.08	-0.01	0.11	-0.09	0.29	0.13	0.13	0.16	-0.14	-0.20	1		
**17. Exhaustion (T2)**	-0.19	0.39	0.37	-0.27	-0.11	-0.17	0.37	-0.04	0.46	-0.17	-0.21	-0.20	-0.21	0.63	0.44	-0.07	1	
**18. Cynicism (T2)**	-0.08	0.40	0.32	-0.34	-0.17	-0.23	0.26	-0.05	0.36	-0.24	-0.22	-0.28	-0.23	0.47	0.60	-0.19	0.60	1
**19. Professional efficacy (T2)**	0.03	-0.12	-0.03	0.18	0.12	0.11	-0.04	0.10	-0.17	0.24	0.11	0.16	0.18	-0.21	-0.26	0.51	-0.18	-0.24

Supplementary Table S2. Fully adjusted linear regression models of the associations between Effort-Reward Imbalance (ERI) dimensions, effort-reward ratio, and over-commitment measured at the first assessment (2014-2018) and the scores of burnout dimensions measured at the second assessment (2018-2021) adjusting for potential covariates (n=575)

	Exhaustion<br>β<br>(95% CI)	Cynicism<br>β<br>(95% CI)	Professional efficacy<br>β<br>(95% CI)
Effort	0.00<br>(-0.09, 0.10)	0.07<br>(-0.02, 0.17)	0.08<br>(-0.02, 0.18)
Reward	0.03	0.08	0.01
Esteem	(-0.05, 0.10)	(-0.00, 0.16)	(-0.07, 0.09)
Security	0.01<br>(-0.07, 0.09)	-0.07<br>(-0.15, 0.01)	0.02<br>(-0.07, 0.10)
Promotion	-0.04<br>(-0.12, 0.03)	-0.06<br>(-0.14, 0.02)	-0.00<br>(-0.08, 0.08)
Over-commitment	0.02<br>(-0.06, 0.11)	-0.04<br>(-0.13, 0.05)	0.03<br>(-0.06, 0.13)
Age	**-0.12**<br>**(-0.19 , -0.05)**	0.01<br>(-0.06, 0.09)	-0.00<br>(-0.08, 0.07)
Sex	-0.05<br>(-0.20 , 0.10)	0.12<br>(-0.04, 0.28)	0.11<br>(-0.05, 0.27)
Length of follow-up	-0.06<br>(-0.13, 0.01)	-0.02<br>(-0.09, 0.06)	0.03<br>(-0.05, 0.10)
Smoking status			
Non smokers	Reference		
Active smokers	0.12<br>(-0.01, 0.29)	0.09<br>(-0.07, 0.25)	-0.05<br>(-0.21, 0.11)
Former smokers	0.07<br>(-0.18, 0.32)	-0.07<br>(-0.33, 0.19)	-0.07<br>(-0.34, 0.19)
Physical activity [a]	-0.15<br>(-0.02, 0.32)	**-0.22**<br>**(0.03, 0.40)**	0.04<br>(-0.23, 0.14)
Burnout at T1			
Exhaustion	**0.42**<br>**(0.32, 0.53)**	0.08<br>(-0.02, 0.19)	-0.04<br>(-0.15, 0.07)
Cynicism	0.02<br>(-0.08, 0.11)	**0.32**<br>**(0.22, 0.41)**	**-0.16**<br>**(-0.25, -0.06)**
Professional efficacy	0.04<br>(-0.03, 0.11)	-0.04<br>(-0.11, 0.04)	**0.42**<br>**(0.34, 0.49)**
Major Depressive Disorder			
Never depressed	Reference		
Current	0.02<br>(-0.13, 018)	0.00<br>(-0.16, 0.17)	-0.00<br>(-0.16, 0.16)
Recovered	-0.23<br>(-0.53, 0.06)	0.05<br>(-0.26, 0.36)	0.01<br>(-0.30, 0.33)
Illicit drug use disorder (abuse/dependence)			
Never	Reference		
Current	0.15	0.08	0.18

	(-0.09, 0.39)	(-0.18, 0.33)	(-0.08, 0.44)
Recovered	-0.64	-0.42	0.39
	(-0.182, 0.53)	(-1.66, 0.82)	(-0.86, 1.65)
Alcohol use disorder (abuse/dependence)			
Never	Reference		
Current	0.07	0.18	-0.06
	(-0.15, 0.29)	(-0.05, 0.41)	(-0.29, 0.18)
Recovered	0.14	-0.15	0.26
	(-0.28, 0.55)	(-0.59, 0.28)	(-0.18, 0.71)
Anxiety disorders [b]			
Never	Reference		
Current	0.05	-0.08	-0.04
	(-0.13, 0.22)	(-0.26, 0.10)	(-0.22, 0.15)
Recovered	-0.26	-0.07	0.19
	(-0.65, 0.14)	(-0.49, 0.35)	(-0.24, 0.62)
Personality traits			
Neuroticism	**0.16**	0.07	-0.06
	**(0.07, 0.25)**	(-0.02, 0.17)	(-0.16, 0.03)
Extraversion	-0.07	**-0.09**	0.04
	(-0.15, 0.00)	**(-0.17, -0.01)**	(-0.04, 0.12)
Social support			
From family	0.02	0.00	-0.08
	(-0.06, 0.10)	(-0.08, 0.09)	(-0.17, 0.01)
From friends	0.01	-0.06	0.03
	(-0.08, 0.09)	(-0.15, 0.03)	(-0.06, 0.12)
From significant others	-0.08	-0.04	0.09
	(-0.17, 0.01)	(-0.13, 0.05)	(-0.01, 0.17)
Adjusted R^2	*0.34*	*0.28*	*0.26*

[a] at least once a week
[b] generalized anxiety disorder, panic disorder, agoraphobia, social phobia
